# NO-Dependent Mechanisms of Myosin Heavy Chain Transcription Regulation in Rat Soleus Muscle After 7-Days Hindlimb Unloading

**DOI:** 10.3389/fphys.2020.00814

**Published:** 2020-07-10

**Authors:** Kristina A. Sharlo, Inna I. Paramonova, Irina D. Lvova, Natalia A. Vilchinskaya, Anna E. Bugrova, Tatiana F. Shevchenko, Grigoriy R. Kalamkarov, Boris S. Shenkman

**Affiliations:** ^1^Myology Laboratory, Institute of Biomedical Problems, Russian Academy of Sciences, Moscow, Russia; ^2^Neurochemistry Laboratory, Emanuel Institute of Biochemical Physics, Russian Academy of Sciences, Moscow, Russia

**Keywords:** NFATc1, myosin isoforms, hindlimb unloading, NO, postural muscle

## Abstract

It is known that nitric oxide (NO) may affect myosin heavy chain (MyHC) isoform mRNA transcription in skeletal muscles. The content of NO in soleus muscles decreases during rat hindlimb unloading as well as slow MyHC mRNA transcription. We aimed to detect which signaling pathways are involved in NO-dependent prevention of hindlimb-suspension (HS)-induced changes in MyHCs’ expression pattern. Male Wistar rats were divided into four groups: cage control group (C), hindlimb suspended for 7 days (7HS), hindlimb suspended for 7 days with L-arginine administration (7HS+A) (500 mg/kg body mass), and hindlimb suspended for 7 days with both L-arginine (500 mg/kg) and NO-synthase inhibitor L-NAME administration (50 mg/kg) (7HS+A+N). L-arginine treatment during 7 days of rat HS prevented HS-induced NO content decrease and slow MyHC mRNA transcription decrease and attenuated fast MyHC IIb mRNA transcription increase; it also prevented NFATc1 nuclear content decrease, calsarcin-2 expression increase, and GSK-3β Ser 9 phosphorylation decrease. Moreover, L-arginine administration prevented the HS-induced *myh7b* and PGC1α mRNAs content decreases and slow-type genes repressor SOX6 mRNA transcription increase. All these slow fiber-type protective effects of L-arginine were blocked in HS+A+N group, indicating that these effects were NO-dependent. Thus, NO decrease prevention during HS restores calcineurin/NFATc1 and *myh7b*/SOX6 signaling.

## Introduction

Skeletal muscles are composed of fibers with different functional properties arranged in a mosaic pattern. Slow-type fibers are characterized by a high fatigue resistance, a longer duration of contraction, but also a lower maximum force. Fast-type fibers demonstrate a high contraction force, but an increased fatigability. The predominance of one of these fiber types in a skeletal muscle determines the phenotype of the muscle. During the last decade, it has been discovered that muscle fiber type is determined by the relative content of slow and fast myosin heavy chain (MyHC) isoforms in the fiber (Schiaffino and Reggiani, 2011). Activity-dependent signaling pathways control skeletal muscle phenotype by affecting transcriptional coactivators/corepressors that are responsible for “slow-type” or “fast-type” muscle genes expression (Meissner et al., 2007).

Functional unloading of skeletal muscles causes severe changes in muscle phenotypes and functioning, especially in slow postural muscles such as soleus muscle. Ground-based models of muscle functional unloading or inactivity such as rat hindlimb unloading (suspension) (Stevens et al., 1999a), human bedrest (Shenkman and Kozlovskaya, 2019), head-out water immersion (Shenkman and Kozlovskaya, 2019), and cat spinal cord transection (Talmadge et al., 1996) result in decrease in slow type I MyHC (MyHC I(β)) in postural muscles.

Calcineurin/NFATc1 and HDAC4/MEF-2D are activity-dependent signaling pathways regulating MyHC I(β) mRNA transcription in skeletal muscle by two parallel mechanisms, fine-tuning MyHC I(β) content in response to different physiological stimuli (Shen et al., 2006). Calcineurin (CaN) is a serine/threonine phosphatase: when interacting with the calcium–calmodulin complex, CaN can dephosphorylate NFATc1 (nuclear factor of activated T-cells, cytoplasmic 1) and induce its nuclear translocation (Chin et al., 1998). Inside the nuclei, NFATc1 binds to MyHC I(β) promoter and induces activation of MyHC I(β) transcription, cooperating with other transcriptional activators and coactivators such as p300 and MEF-2D (myocyte enhancer factor 2D) (Meissner et al., 2007). Several protein kinases such as glycogen synthase kinase 3β (GSK-3β) (Beals et al., 1997), p38 MAPK (p38) (Braz et al., 2003), c-Jun N-terminal kinase (JNK) (Liang et al., 2003), and protein kinase A (PKA) (Beals et al., 1997; Sheridan et al., 2002) can phosphorylate NFAT, causing its exit from the nucleus (Wu et al., 2001).

The transcriptional activity of MEF2 is suppressed by members of the family of class II histone deacetylase (HDAC) proteins (Miska et al., 1999). Repression of nuclear MEF-2 by class II HDACs is regulated by the phosphorylation status of HDACs. Activation of several protein kinases such as calcium calmodulin kinase CaMK (McKinsey et al., 2001) and AMPK (Rockl et al., 2007) leads to phosphorylation of HDACs and their export from the nuclei. MEF-2 transcriptional factors were also shown to upregulate the slow-tonic myosin *myh7b* gene (Warkman et al., 2012). The *myh7b* gene encodes an intronic microRNA, miR-499 (van Rooij et al., 2009). Frequently, *myh7b* pre-mRNA undergoes non-productive splicing, resulting in an RNA that does not encode a functional protein while at the same time promoting the expression of miR-499, so that *myh7b* mRNA and miR-499 showed a correlated expression (Bell et al., 2010). MiR-499 is predicted to target the 3′ UTR of the transcriptional repressor SOX6 (SRY-Box Transcription Factor 6), which is involved in the repression of slow fiber type genes, in particular, MyHC I(β) (McCarthy et al., 2009). It was also shown that miR-499 upregulates PGC1α (peroxisome proliferator-activated receptor-gamma coactivator-1α) expression in skeletal muscle fibers, supporting oxidative metabolism of the fiber (Xu et al., 2018). *myh7b* expression may be in turn upregulated by micro-RNA 208-b, encoded by MyHC I (β) gene, forming positive feedback loop for MyHC I(β) transcription (McCarthy et al., 2009). So it may be possible that prevention of the unloading-induced MyHC I(β) mRNA transcription decrease may also prevent *myh7b* and PGC1α expression decreases and SOX6 expression increase, supporting oxidative genes expression.

The main activity-dependent signals that could upregulate both HDAC4/MEF-2D and calcineurin/NFATc1 cascades are calcium ions and nitric oxide (NO). However, the level of intracellular calcium increases after the 2nd day of unloading and remains elevated compared with the control group up to 14th day of unloading, so it is unlikely that unloading-induced MyHC expression decline could be triggered by calcium-dependent mechanisms (Ingalls et al., 2001). The level of NO in soleus muscle decreases during unloading (Lomonosova et al., 2011), and it was observed that NO could contribute to slow MyHC transcription by cGMP-dependent GSK-3β inactivation by Ser 9 phosphorylation (Drenning et al., 2008) and by AMPK activation (Chen et al., 2018a), thereby affecting the both MyHC-regulating signaling cascades. It was shown that prevention of the unloading-induced NO decrease in soleus muscles leads to slow MyHC transcription increase after 2 weeks of unloading (Lomonosova et al., 2011), but the detailed mechanism (or mechanisms) of this effect remains unclear.

So, the present study is aimed to analyze which key signaling pathways regulating slow myosin transcription are involved in NO-dependent prevention of unloading-induced decrease of MyHC I (β) mRNA transcription.

## Materials and Methods

### Ethics Statement

All procedures with the animals were approved by the Biomedicine Ethics Committee of the Institute of Biomedical Problems of the Russian Academy of Sciences/Physiology Section of the Russian Bioethics Committee. All experiments were performed in strict accordance with the guidelines and recommendations in the Guide for the Care and Use of Laboratory Animals of the National Research Council of the National Academies of Sciences. All efforts were made to minimize the animals’ pain and suffering. Animals were housed in a temperature-controlled room on a 12:12-h light-dark cycle with food pellets and water provided *ad libitum*; 3-month-old male Wistar rats were obtained from the certified Nursery for laboratory animals of the Institute of Bioorganic Chemistry of the Russian Academy of Sciences (Pushchino, Moscow region). On completion of the experiment, the animals were sacrificed by i.p. injection of tribromoethanol overdose (750 mg/kg) followed by cervical dislocation. The depth of anesthesia was evaluated by testing the pedal withdrawal reflex.

### Experimental Design

Sixty-four 2.5-month-old male Wistar rats of 180–230 g were assigned to four groups (16 animals in each): cage control (C) group, hindlimb suspended for 7 days (7HS), hindlimb suspended for 7 days with the daily intraperitoneal L-arginine administration (7HS+A) (L-arginine concentration 500 mg/kg rat body mass), and hindlimb suspended for 7 days with both L-arginine (500 mg/kg rat body mass) and L-NAME (N-nitroarginine methyl ester, 50 mg/kg rat body mass) daily intraperitoneal administration (7HS+A+N). All the substances administered intraperitoneally were dissolved in 1.5 ml of 1% dimethylsulfoxide (DMSO) in phosphate-buffered saline (PBS). C and HS groups underwent daily 1.5 ml of 1% DMSO in PBS placebo injections. L-NAME is an NO-synthase inhibitor, so 7HS+A+N group was aimed to detect possible NO-independent signaling effects of L-arginine. The weight of experimental animals did not change significantly during the experiment.

Eight animals from each group underwent intrasoleus injection of the spin trap DETC 30 min before being sacrificed for the detection of NO content in soleus muscles. The soleus muscles of the rest eight animals from each group were used to prepare protein and total RNA fractions. The animals were sacrificed as described above and their *musculus soleus* were frozen in liquid nitrogen. The body weights of the experimental animals did not significantly differ among the groups after the experiment and the soleus muscles weights of all the hindlimb-unloaded animals significantly decreased compared to control (Table 1).

**TABLE 1:** Body weights and soleus weights of the experimental animals.

Group	C	7HS	7HS+A	7HS+A+N
Rat body weight, g (mean, maximum and minimum)	203 (216–186)	197 (205–181)	187 (198–181)	182 (187.5–164.5)
*m soleus* weight, mg (mean, maximum and minimum)	121.7 (115.75–125.25)	73.2* (66.5–74.9)	68.2* (66.45–70.09)	76.1* (64.55–79.15)
*m soleus* weight/body weight, mg/g	0.113 (0.110–0.116)	0.086* (0.081–0.087)	0.099* (0.097–0.100)	0.100* (0.8–0.134)

*Significant differences from C group.

### Hindlimb Suspension Model

Gravitational unloading was simulated using a standard hindlimb suspension (HS) model (Morey-Holton and Globus, 2002). Briefly, a strip of adhesive tape was applied to the animal’s tail, which was suspended by passing the tape through a swivel that was attached to a metal bar on the top of the cage. This allowed the forelimbs to have contact with the grid floor and allowed the animals to move around the cage for free access to food and water. The suspension height was adjusted to prevent the hindlimbs from touching any supporting surface while maintaining a suspension angle of approximately 30°.

### NO Detection

Nitric oxide content in soleus muscles was analyzed as described previously (Lomonosova, 2009). Briefly, relative intramuscular NO content was determined using a standard spin trap and electron paramagnetic resonance technique (EPR). We used diethyldithiocarbamate (DETC) as a spin trap that forms in tissues nitrosyl paramagnetic complexes with iron, which is in equilibrium with stationary NO concentration within the tissue and have a characteristic EPR spectrum. The spin trap was injected into rat soleus muscles at the rate of 500 mg/kg body mass. Immediately after the injection of DETC, aqueous solution of 29 mM FeSO_4_ mixture with 116 mM sodium citrate (2 ml/kg body mass) was injected in rat soleus muscle. After 30 min, the animals were sacrificed as described above and their *m. soleus* were frozen in liquid nitrogen. The EPR signal was registered on a Bruker EMX-8 EPR spectrometer.

### Nuclear and Cytoplasmic Extracts Preparation

Nuclear extracts were prepared from 50 mg of soleus muscle using NE-PER Nuclear and Cytoplasmic Extraction Reagents (Thermo Fisher Scientific, United States). Complete Protease Inhibitor Cocktail (Santa Cruz), Phosphatase Inhibitor Cocktail B (Santa Cruz), PMSF (1 mM), aprotinin (10 μg/ml), leupeptin (10 μg/ml), and pepstatin A (10 μg/ml) were used to maintain extract integrity and function. Nuclear extracts were dialyzed by means of Amicon Ultra-0.5 centrifuge filters (Millipore, United States).

The protein content of all samples was quantified twice using a Quick Start Bradford Protein Assay (Bio-Rad Laboratories) in order to calculate the optimal sample value for electrophoretic gel. The supernatant fluid was diluted with 2× sample buffer (5.4 mM Tris–HCl, pH 6.8, 4% SDS, 20% glycerol, 10% β-mercaptoethanol, 0.02% bromophenol blue) and stored at −85°C for immunoblot procedures. The quality of nuclear and cytoplasmic fractions separation was evaluated by performing GAPDH immunoblot detection in nuclear samples and lamin B1 immunoblot detection in cytoplasmic samples: no bands were detected in each case.

### Total Protein Fraction Preparation

Total muscle protein fractions were prepared from 20 mg of soleus muscle tissue cryosections, homogenized for 25 min in 100 μl buffer RIPA Lysis Buffer System (Santa Cruz, United States) with addition of 10 mM EDTA, 50 mM β-glycerophosphate, 0.5 mM DTT, 10 μg/ml aprotinin, 10 μg/ml leupeptin, 1 mM PMSF, 50 mM NaF, 1 mM Na3VO4, 10 μg/ml pepstatin, and 20 μl “complete Mini Protease Inhibitor Cocktail.” Then, the muscle lysate samples were centrifuged at 20,000 *g* for 25 min. The protein content of all samples was quantified twice as described for nuclear and cytoplasmic fractions, diluted in 2× sample buffer and stored at −85°C.

### Immunoblots

The contents of NFATc1 and MEF-2D were analyzed in nuclear protein fraction, p/total GSK-3β content was analyzed in total protein fraction, and p/total AMPK and calsarcin-2 were analyzed in cytoplasmic fraction. For each protein analyzed, conditions of electrophoresis and Western blotting were optimized, according to the protein molecular weight and quantity in the lysate. Electrophoresis was carried out in the 10% separating polyacrylamide gel (0.2% methylene-bisacrylamide, 0.1% SDS, 375 mM Tris-HCl, pH 8.8, 0.05% ammonium persulfate, 0.1% TEMED) and in the 5% concentrating polyacrylamide gel (0.2% methylene-bisacrylamide, 0.1% SDS, 125 mM Tris–HCl, pH 6.8, 0.05% ammonium persulfate, 0.1% TEMED). The cathode (192 mM Tris-glycine, pH 8.6, 0.1% SDS) and anode (25 mM Tris–HCl, pH 8.6) buffers were used. Samples were loaded at the rate of 25 μg of total protein in each sample. The samples of each group were loaded on the gel together with control samples. Electrophoresis was carried out at 17 mA/gel in a mini system (Bio-Rad Laboratories) at room temperature.

Electrotransfer of the proteins was carried out in buffer (25 mM Tris, pH 8.3, 192 mM glycine, 20% ethanol, 0.04% SDS) onto nitrocellulose membrane at 100 V and 4°C in the mini Trans-Blot system (Bio-Rad) for 120 min. The membranes were blocked in 5% non-fat dry milk solution (Bio-Rad) in PBST (PBS pH 7.4, 0.1% Tween 20) for 1 h at room temperature. To reveal protein bands, the following primary polyclonal antibodies were used: total GSK-3β and phosphorylated Ser 9 GSK-3β (Cell Signaling, 1: 1,000), GAPDH (Cell Signaling, 1: 10,000), lamin B1 (Abcam, 1:1,000), calsarcin-2 (Proteintech, 1:3,000), phospho-AMPK, total AMPK, MEF-2D (1:1000 EMD Millipore), phosphorylated Thr 172 AMPKα1/2 (Santa Cruz Biotechnology, 1:500), and total AMPK α1/2 (Cell Signaling, 1:500). All the primary antibodies were used for overnight incubation at 4°C. The secondary HRP-conjugated antibodies (goat-anti-rabbit, Santa Cruz, 1: 30,000, goat-anti-mouse, Santa Cruz, 1: 25,000) were used for 1 h incubation at room temperature. The blots were washed three times, 10 min each, in PBST. Then, the blots were revealed using the ImmunStar TM Substrate Kit (Bio-Rad Laboratories, United States) and the C-DiGit Blot Scanner (LI-COR Biotechnology, United States). Protein bands were analyzed using the Image Studio Digits Ver. 4.0 software. All image densities were measured in linear range of scanner detection. The optical absorption (OA) of the control group band on analytical membrane was taken as 100%, while the OA of other groups was compared with that of the control group bands localized on the same membrane. The blots on which phosphorylated proteins were detected were stripped with Restore Western Blot Stripping Buffer (Thermo Fisher Scientific) and then re-probed with total protein antibodies overnight at 4°C to analyze the phosphorylation level of the proteins. Then, the blots were incubated with HRP-conjugated goat-anti-rabbit secondary antibody and visualized as described above. It was controlled that phosphorylated proteins–goat-anti-rabbit-HRP complexes were washed out completely from the blots. The blots were washed 3 × 10 min at room temperature with PBST after incubations with antibodies and Restore Western Blot Stripping Buffer. The signals of all protein bands from total protein fraction except for phosphorylated proteins were normalized to GAPDH; phosphorylated proteins were normalized to total proteins content. Nuclear fraction proteins’ signals were normalized to lamin B1. Moreover, Ponceau S staining of the membranes was utilized to ensure equal loading of the extracts (not shown). All Western blots were repeated at least three times.

### RNA Isolation

Total RNA was extracted from frozen soleus muscle samples using RNeasy Micro Kit (Qiagen, Germany) according to the manufacturer’s protocol. RNA concentration was analyzed at 260 nm. RNA quality of purification was evaluated according to 260/280 and 260/230 ratios, and its integrity was assessed by gel electrophoresis with ethidium bromide staining of 1 μg total RNA on 1 % agarose gel. The RNA solutions were stored frozen at −85°C and were used in RT-PCR procedures.

### RT-qPCR

Reverse transcription was performed by incubation of 0.5 μg of RNA, random hexamers d(N)6, dNTPs, RNase inhibitor, and MMLV reverse transcriptase for 60 min at 42°C. The samples to be compared were run under similar conditions (template amounts, duration of PCR cycles). The annealing temperature was based on the PCR primers’ optimal annealing temperature. PCR primers used for RNA analysis are shown in Table 2. The amplification was real-time monitored using SYBR Green I dye and the iQ5 Multicolor Real-Time PCR Detection System (Bio-Rad Laboratories, United States). To confirm the amplification specificity, the PCR products from each primer pair were subjected to a melting curve analysis, and sequencing of the products was provided at least once. Relative quantification was performed based on the threshold cycle (CT value) for each of the PCR samples (Livak and Schmittgen, 2001). *RPL19* was tested and chosen for the normalization of all quantitative PCR analysis experiments in the current study.

**TABLE 2:** Primers used for RT-qPCR.

Gene description	Primer sequence	Reference sequence
*Myh7* (MyHC I(β))	5′-ACAGAGGAAGACAGGAAGAACCTAC-3′ 5′-GGGCTTCACAGGCATCCTTAG-3′	*NM_017240.2*
*Myh7b*	5′-GAGTGTGGAGCAGGTGGTATTT-3′ *5*′*-GGACCCCAATGAAGAACTGA-3*′	*NM_001107794.2*
*Myh2* (MyHCIIa)	5′-TATCCTCAGGCTTCAAGATTTG-3′ 5′-TAAATAGAATCACATGGGGACA-3′	NM_001135157.1
*Myh4* (MyHCIIb)	5′-CTGAGGAACAATCCAACGTC-3′ 5′-TTGTGTGATTTCTTCTGTCACCT-3′	NM_019325.1
*Myh1* (MyHCIId/x)	5′-CGCGAGGTTCACACCAAA-3′ 5′-TCCCAAAGTCGTAAGTACAAAATGG-3′	NM_001135158.1
*SOX6*	5′-TCAAAGGCGATTTACCAGTGAC-3′ 5′-TTGTTGTGCATTATGGGGTGC-3′	*NM_001024751.1*
*Rcan1 (MCIP1.4)*	5′-CCGTTGGCTGGAAACAAG-3′ 5′-GGTCACTCTCACACACGTGG-3′	NM_153724.2
RPL19	5′- GTACCCTTCCTCTTCCCTATGC-3′ *5*′*- CAATGCCAACTCTCGTCAACAG-3′*	NM_031103.1
*PGC1*α (PPARG coactivator 1 alpha)	5′-GTGCAGCCAAGACTCTGTATGG-3′ *5′-* GTCCAGGTCATTCACATCAAGTTC*-3′*	NM_031347.1
*Myoz1* (Calsarcin-2)	5′-GTGGAACTTGGCATTGACCT-3′ 5′-GAGGACCAAGGGTTCACTCA-3′	NM_001109097.1

### Statistical Analysis

All values are shown as means ± SEM of eight animals. The means of all groups are shown as % of the control group. To check whether the differences among groups were statistically significant, given the small sample sizes and comparisons between three groups, we adopted the Kruskal–Wallis nonparametric test, which is statistically informative despite the small number of subjects in each group, followed by Dunn’s *post hoc* test for the comparisons between four groups. A *p*-value less than 0.05 was regarded as statistically significant.

## Results

### Detection of NO Content in Soleus Muscle

**FIGURE 1:**
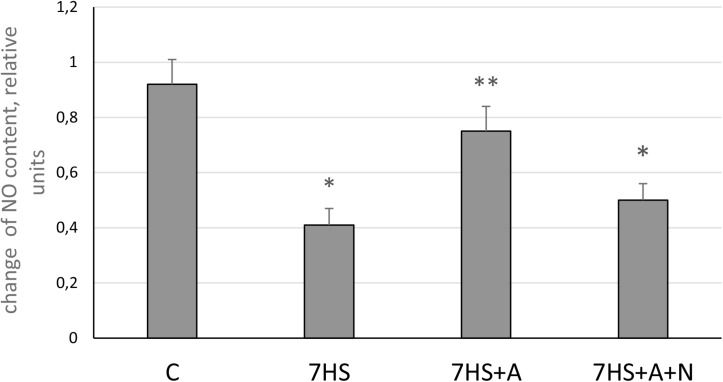
Nitric oxide content in cage control group (C), hindlimb suspended for 7 days group (7HS), hindlimb suspended for 7 days L-arginine administered group (7HS+A), and hindlimb suspended for 7 days with both L-arginine and L-NAME administration (7HS+A+N). There are eight animals in each group. All data are shown as % of control group (mean ± SEM). * Significant differences from control groups (*p* < 0.05); ** significant differences from 7HS.

The level of NO in soleus muscles of hindlimb-unloaded animals (7HS) was significantly decreased by 56% compared to control (Figure 1). In L-arginine administered hindlimb-unloaded group (7HS+A), the content of NO was significantly higher than in 7HS group and was equal to the control group value. In the unloaded group with both L-arginine and L-NAME administration (7HS+A+N), NO content was significantly decreased by 46% compared to control.

### MyHCs mRNA Transcription

**FIGURE 2:**
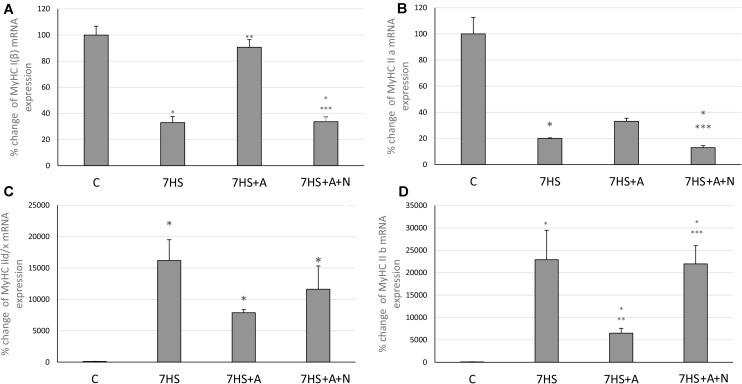
Cage control group (C), hindlimb suspended for 7 days group (7HS), hindlimb suspended for 7 days L-arginine administered group (7HS+A), hindlimb suspended for 7 days with both L-arginine and L-NAME administration group (7HS+A+N). There are eight animals in each group. **(A)** MyHC Iβ mRNA transcription, **(B)** MyHC IIa mRNA transcription, **(C)** MyHC II d/x mRNA transcription, and **(D)** MyHC II b mRNA transcription. All data are shown as % of control groups (mean ± SEM). * Significant differences from control group (*p* < 0.05); ** significant differences from 7HS; *** significant differences from 7HS+A.

The transcription of slow MyHC I and fast MyHC IIa mRNAs after 7 days of hindlimb suspension (7HS) significantly decreased compared to control group (Figures 2A,B), and fast MyHCs II d/x and II B mRNAs transcription significantly increased (Figures 2C,D). L-arginine administration prevented unloading-induced MyHC I mRNA transcription decrease and attenuated MyHC IIa mRNA transcription decrease as well as MyHC II B transcription increase (Figure 2). However, L-arginine administration did not affect MyHC II d/x mRNA transcription increase. L-NAME blocked all these effects of L-arginine in group 7HS+A+N, so we can conclude that L-arginine prevents unloading-induced MyHC I and MyHC IIa mRNAs transcription decreases by affecting the content of NO in soleus muscles of unloaded animals.

### Calcineurin/NFATc1 Signaling

**FIGURE 3:**
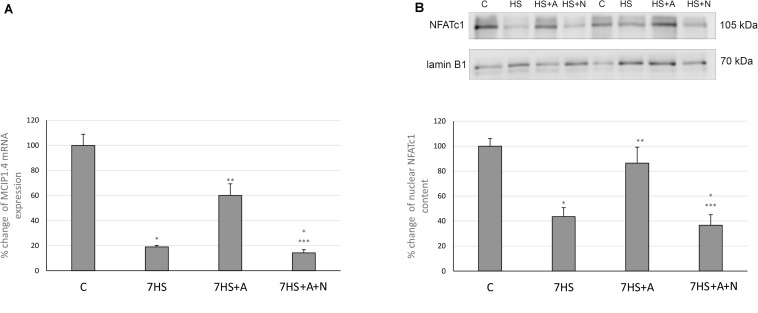
Cage control group (C), hindlimb suspended for 7 days group (7HS), hindlimb suspended for 7 days L-arginine administered group (7HS+A), hindlimb suspended for 7 days with both L-arginine and L-NAME administration group (7HS+A+N). There are eight animals in each group. **(A)** MCIP 1.4 mRNA transcription, **(B)** NFATc1 nuclear content. All data are shown as % of control groups (mean ± SEM). * Significant differences from control group (*p* < 0.05); ** significant differences from 7HS; *** significant differences from 7HS+A.

**FIGURE 4:**
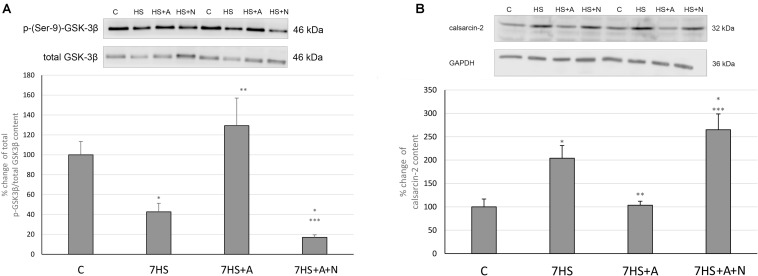
Cage control group (C), hindlimb suspended for 7 days group (7HS), hindlimb suspended for 7 days L-arginine administered group (7HS+A), hindlimb suspended for 7 days with both L-arginine and L-NAME administration group (7HS+A+N). There are eight animals in each group. **(A)** % of Ser 9 phosphorylation of GSK-3 β in total protein fraction; **(B)** calsarcin-2 content in total protein fraction. All data are shown as % of control groups (mean ± SEM). * significant differences from control group in all the mentioned figures; ** Significant differences from 7HS; *** significant differences from 7HS+A.

Modulatory calcineurin-interacting protein 1 (MCIP1.4) mRNA transcription, which is used as the marker of NFAT-dependent transcriptional activity (Rothermel et al., 2000; Yang et al., 2000), significantly decreased in 7HS group (Figure 3A). L-arginine administration partially prevented this decrease in 7HS+A group so that the decrease in MCIP1.4 mRNA transcription in this group was not statistically significant. In 7HS+A+N group, the MCIP1.4 mRNA transcription decrease was similar to the decrease in 7HS group. NFATc1 nuclear content was significantly decreased in 7HS and 7HS+A+N groups, and L-arginine administration prevented NFATc1 nuclear content decrease in group 7HS+A, so NO content increase can prevent NFAT-dependent transcription decrease and NFATc1 myonuclear content decrease after 7 days of hindlimb unloading (Figure 3B). The percent of Ser 9 phosphorylated GSK-3β in total protein fraction decreased by 58% in 7HS group and by 78% 7HS+A+N group, showing increased GSK-3β activity (Figure 4A). In 7HS+A group, Ser 9 phosphorylated GSK-3β was 129% from control and did not statistically differ from C group. The activity of calcineurin in skeletal muscle can be inhibited by endogenous calcineurin inhibitor calsarcin-2 (FATZ-1, myosenin-1) (Frey et al., 2000). The expression of calsarcin-2 mRNA and protein increased more than twofold in all the hindlimb unloaded groups except for 7HS+A group, in which calsarcin-2 expression did not differ from the control group (Figure 4B). So, we can conclude that calsarcin-2 expression in unloaded soleus muscles may be regulated by NO.

### AMPK/MEF-2D Signaling

**FIGURE 5:**
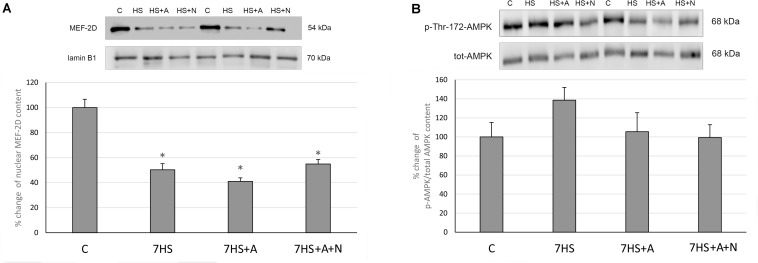
Cage control group (C), hindlimb suspended for 7 days group (7HS), hindlimb suspended for 7 days L-arginine administered group (7HS+A), hindlimb suspended for 7 days with both L-arginine and L-NAME administration group (7HS+A+N). There are eight animals in each group. **(A)** MEF-2D content in nuclear fraction; **(B)** % of Thr-172 phosphorylation of AMPKα1/2 in cytoplasmic fraction. All data are shown as % of control groups (mean ± SEM). * Significant differences from control group (*p* < 0.05).

MEF-2D nuclear content was significantly decreased in all the hindlimb unloaded groups: L-arginine administration could not prevent this decrease (Figure 5A). Surprisingly, we did not observe any effect of L-arginine administration on the percent of AMPK Thr-172 phosphorylation (Figure 5B), so we can conclude that the effect of L-arginine administration on MyHC isoforms mRNA transcription in unloaded rat soleus muscles is not mediated by AMPK phosphorylation and may be independent of AMPK activity.

### SOX6*/myh7b* Signaling

**FIGURE 6:**
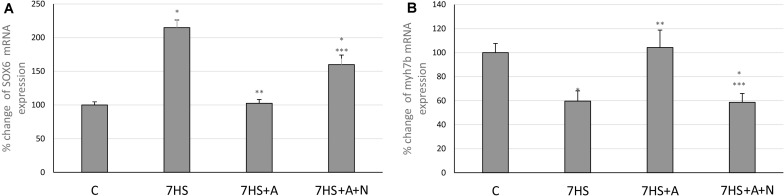
Cage control group (C), hindlimb suspended for 7 days group (7HS), hindlimb suspended for 7 days L-arginine administered group (7HS+A), hindlimb suspended for 7 days with both L-arginine and L-NAME administration group (7HS+A+N). There are eight animals in each group. **(A)** myh7b mRNA transcription; **(B)** SOX6 mRNA transcription. All data are shown as % of control groups (mean ± SEM). * significant differences from control group in all the mentioned figures; ** Significant differences from 7HS; *** significant differences from 7HS+A.

**FIGURE 7:**
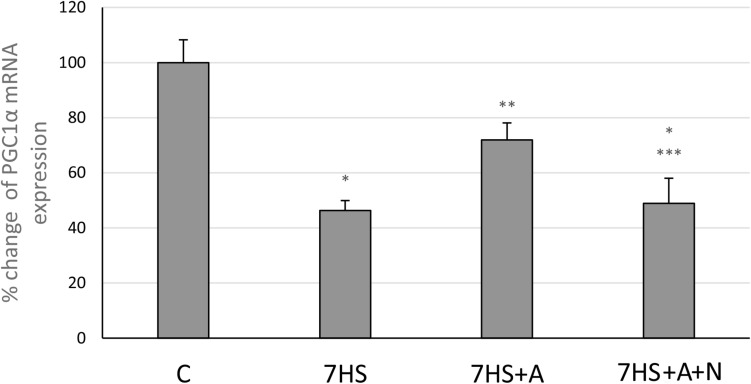
Cage control group (C), hindlimb suspended for 7 days group (7HS), hindlimb suspended for 7 days L-arginine administered group (7HS+A), hindlimb suspended for 7 days with both L-arginine and L-NAME administration group (7HS+A+N). There are eight animals in each group. PGC1α mRNA transcription. All data are shown as % of control groups (mean ± SEM). * significant differences from control group in all the mentioned figures; ** significant differences from 7HS; *** significant differences from 7HS+A.

After 7 days of hindlimb unloading the expression of *myh7b* gene mRNA significantly decreased (Figure 6A), while the transcription of SOX6 mRNA increased twofold (Figure 6B). The transcription of *myh7b* gene mRNA as well as the transcription of SOX6 mRNA returned to the control group level in 7HS+A group, while in 7HS+A+N group the transcription of SOX6 and *myh7b* gene mRNAs was similar to 7HS group (Figures 6A,B). The level of mRNA transcription of PGC1α was significantly decreased in 7HS group. L-arginine administration prevented this decrease, and L-NAME administration blocked this effect of L-arginine (Figure 7).

## Discussion

The observed NO content decrease in 7HS as well as its increase in L-arginine administered group (Figure 1) correspond to our previous data (Lomonosova et al., 2011). These data also correspond to the total nNOS content decrease in sarcolemma during 10 days and 14 days of gravitational unloading (Tidball et al., 1998), prolonged bed-rest or immersion of human volunteers (Rudnick et al., 2004; Suzuki et al., 2007), and the data of nNOS mRNA transcription decrease during 14 days of unloading (Lomonosova et al., 2011). However, it should be noticed that Suzuki et al. (2007) showed NO increase during 14 days of mice unloading, which is at variance with our data. At the same time, in the study by Suzuki et al. (2007), the levels of NO in the nNOS knockout mice and in the mice treated with nNOS inhibitor were the same as in control wild-type mice. Suzuki and colleagues explained the increase of NO concentration in muscle fibers in their experiments by a translocation of nNOS from the membrane to the cytoplasm. This phenomenon was observed on multiple occasions (Suzuki et al., 2007; Lechado et al., 2018), but currently there are no published data documenting higher NO production by non-membrane-bound NOS. Since both studies (Suzuki et al., 2007; Lomonosova et al., 2011) used the same experimental approach in measuring of NO levels, the observed discrepancies need further examination. An important point that should be mentioned is that in our experiment the soleus muscles of the experimental animals were frozen in liquid nitrogen immediately after dissection, and all the subsequent procedures including EPR spectrum recording were conducted at the temperature of liquid nitrogen [similar to the procedure described in Lomonosova et al. (2014)]. Suzuki et al. (2007) minced the soleus muscle tissue and measured NO content at room temperature and they did not mention whether they had blocked the activity of NOS isoforms before the mincing. It was shown that NO production in skeletal muscles is very sensitive to mechanical stimuli (Palomero et al., 2012), so the results of Suzuki may be affected by NO generation during the mincing. However, the differences between spin traps used could also play a role, as we used DETC and Suzuki and colleagues used MGD spin trap.

The MyHC IIb and MHC IId/x mRNAs transcription increases, as well as MyHC IIa and MyHC I(β) mRNAs transcription decreases in the 7HS group are in accordance with many previously obtained data (Stevens et al., 1999b; Giger et al., 2009; Baldwin et al., 2013; Lomonosova et al., 2016). The prevention of MyHC I(β) mRNA transcription decrease in the L-arginine administered group as well as blocking of this L-arginine effect by the nNOS inhibition correspond to the results of various authors, who showed L-arginine dependent upregulation of MyHC I(β) mRNA and the increase of type I skeletal muscle fibers in myotubes and during rodent HS (Drenning et al., 2008; Lomonosova et al., 2011; Chen et al., 2018a, b) and the negative effect of nNOS inhibition on MyHC I(β) expression (Martins et al., 2012; Suwa et al., 2015). On the contrary, Vitadello and co-authors showed that nNOS inhibition by 7-nitroindazole during rat hindlimb unloading did not affect slow-to-fast fiber-type ratio in rat soleus muscles; however, in that study the level of NO in soleus muscle tissue was not shown as well as the level of MyHC I(β) mRNA, so the contradiction may be the result of dose-dependent inhibitor effects or of variability of slow-to-fast fiber-type transition time courses (Vitadello et al., 2014). The attenuated increase of MyHC IIb mRNA in HS+A group may be caused by increased NFAT transcriptional activity, as it was shown that NFATc1 can repress MyHC IIb transcription (McCullagh et al., 2004). Partial prevention of MyHC IIa mRNA transcription decrease in HS+A group may be caused by NO-dependent GSK-3β inactivation, as it was shown that GSK-3β inhibition upregulates MyHC IIa in goat satellite cells (Wang et al., 2019).

The effect of NO on GSK-3β phosphorylation and NFATc1 myonuclear accumulation was previously shown in mouse myotubes by Drenning and co-authors. In their study, it was shown that nNOS activity-mediated Ser 9 phosphorylation of GSK-3β leads to GSK-3β inhibition and NFAT nuclear accumulation (Drenning et al., 2008). The results of our experiment evidence that the restoration of NO content in unloaded soleus muscles leads to Ser 9 phosphorylation of GSK-3β and prevents NFATc1 myonuclear content decrease, so we can assume that this signaling pathway is likely to contribute to the NO-dependent slow MyHC transcription increase during hindlimb unloading. The observed NFATc1 nuclear content decrease is in accordance with our previously obtained data about 1 day HS, although it was shown that NFATc1 nuclear content in soleus muscle fibers does not differ from control group or even exceeds control group values after 3 days and 2 weeks of rat HS (Dupont-Versteegden et al., 2002; Xia et al., 2016; Sharlo et al., 2019). The observed MCIP1.4 mRNA transcription decrease after 1 week of hindlimb unloading is in accordance with our previously obtained data about 1 day HS and 3 days HS (Sharlo et al., 2019). The intragenic region of the MCIP1 gene contains an alternative calcineurin-responsive promoter with NFAT binding motifs near the exon 4 of the gene, so the level of MCIP1.4 mRNA represents the general level of active NFATc1 in the samples (Rothermel et al., 2000, 2001; Yang et al., 2000). So, we can assume that the decrease of nuclear NFATc1 content after 7 days of hindlimb unloading leads to the decrease of NFAT-dependent transcription and that both are prevented by L-arginine administration in an NO-dependent manner.

The increase of the endogenous calcineurin inhibitor calsarcin-2 content after 7 days of hindlimb unloading corresponds to our previous data (Lomonosova et al., 2016). However, we have become the first to show that calsarcin-2 content in unloaded rat soleus is regulated by NO, as now we showed that calsarcin-2 content is equal to control in the 7HS+A group. So it may be possible that protective effect of NO on calcineurin/NFATc1 signaling is mediated by both GSK-3β downregulation and blocking the expression of calcineurin inhibitor calsarcin-2.

It was previously shown that NO contributes to slow MyHC transcription by AMPK activation in myotubes (Chen et al., 2018b), so we suggested that NO content restoration during rat hindlimb unloading would activate AMPK in rat soleus muscles. However, according to the results of our experiment L-arginine administration did not lead to AMPK activation. So we can assume that the protective effect of L-arginine on slow MyHC transcription in soleus muscles does not depend on AMPK phosphorylation. The decrease of nuclear MEF-2D during HS is observed for the first time, and the reasons of this decrease remain unclear. It has been shown that MEF-2D nuclear translocation in skeletal muscle fibers can be induced by AMPK activator AICAR (Holmes et al., 2005). However, our data indicate that the unloading-induced MEF-2D nuclear decrease does not correlate with AMPK phosphorylation or NO content.

**FIGURE 8:**
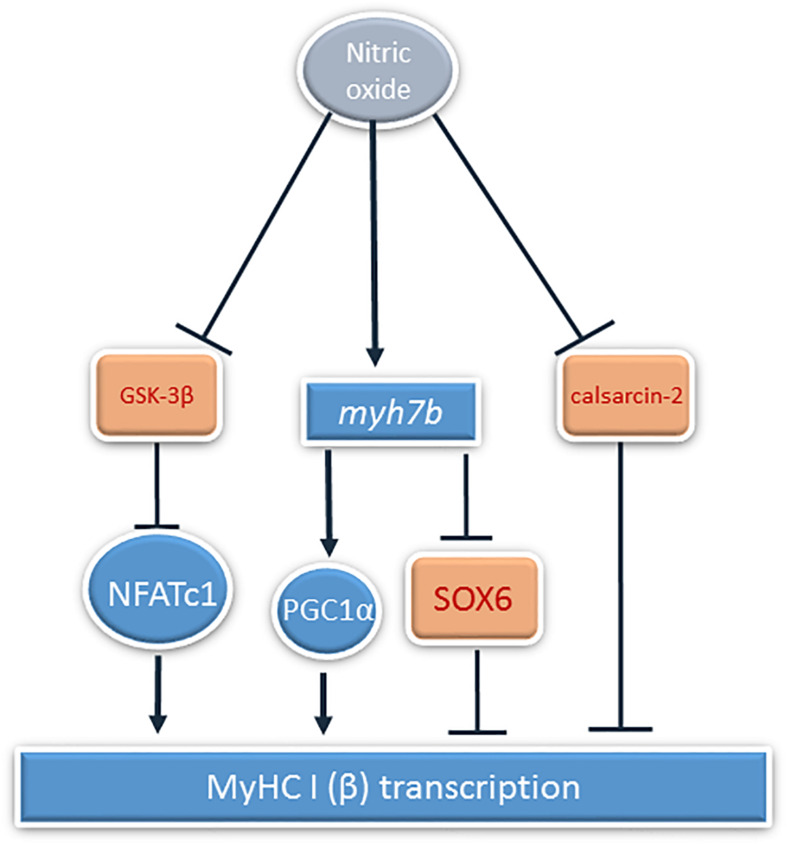
The summary of the NO-dependent mechanisms of MyHC Iβ transcription regulation during rat hindlimb unloading. Activation is shown by pointed arrows, and inhibition is showed by block arrows.

MyHC I(β) mRNA in skeletal muscle fibers induces the transcription of slow-tonic myosin *myh7b* gene by micro-RNA-dependent mechanisms (Bell et al., 2010). *myh7b* mRNA does not translate into a protein molecule, but produces micro-RNA 499 (McCarthy et al., 2009). This micro-RNA targets the 3′ UTR of the transcriptional repressor SOX6, which is involved in the repression of slow fiber type genes, in particular, MyHC I(β), and downregulates SOX6 mRNA transcription (Hagiwara et al., 2005). Based on these facts, we decided to analyze the contents of *myh7b* and SOX6 mRNAs in soleus muscles of the experimental animals. The observed decrease in *myh7b* gene mRNA transcription as well as SOX6 mRNA transcription increase after 7 days of HS correspond to the data of McCarthy et al. (2009), who observed MiR-499 transcription decrease at this time-point. We showed that L-arginine administration during 7 days of unloading led to the restoration of MyHC I(β) and *myh7b* gene mRNAs transcription and SOX6 mRNA downregulation. Despite the fact that *myh7b* gene mRNA transcription depends on MEF-2D, L-arginine supplementation during HS managed to restore *myh7b* gene mRNA content in HS-subjected rat soleus muscles without affecting MEF-2D nuclear content (Warkman et al., 2012). Moreover, L-arginine administration led to the restoration of PGC-1α mRNA transcription. These data correspond to the previously obtained data that NO can upregulate PGC-1α signaling (Baldelli et al., 2014; Theeuwes et al., 2017). However, the effect of NO on PGC-1α transcription is often explained by NO-dependent AMPK phosphorylation, although in our experiments NO did not affect AMPK phosphorylation. Based on this fact, we suggest that PGC-1α mRNA transcription upregulation in 7HS+A group is associated with MyHC I(β)/*myh7b*/MiR-499 signaling pathway upregulation, as MiR-499 was shown to upregulate PGC-1α expression (Xu et al., 2018). Another mechanism of PGC-1α mRNA transcription upregulation in HS+A group can be GSK-3β-dependent, as it was shown that GSK-3β inhibition may lead to PGC-1α mRNA transcription upregulation. It was shown that PGC-1α itself may coactivate NFAT-dependent transcription and upregulate slow MyHC expression (Lin et al., 2002; Handschin et al., 2007), so NO-dependent restoration of PGC-1α mRNA transcription in our experiment may also contribute to the restoration of MyHC I(β) transcription. Thus, the observed MyHC I(β) mRNA transcription increase in L-arginine-treated hindlimb suspended group compared to HS group may be caused by inactivation of GSK-3β and downregulation of calsarcin-2 expression, leading to increased NFATc1 myonuclear accumulation and as well by *myh7b* mRNA upregulation, leading to SOX6 repression. The summary of the results is shown on Figure 8.

It is concluded that prevention of HS-induced NO decrease in rat soleus muscles after 7 days of unloading leads to partial prevention of slow oxidative MyHC I(β) and fast oxidative MyHC IIa mRNAs transcription decreases, supports NFATc1 transcriptional activity, and leads to upregulating *myh7b* gene mRNA and restoration of PGC1α mRNA transcription, counteracting unloading-induced transcriptional alterations.

## Data Availability Statement

The datasets generated for this study are available on request to the corresponding author.

## Ethics Statement

The animal study was reviewed and approved by Biomedicine Ethics Committee of the Institute of Biomedical Problems of the Russian Academy of Sciences/Physiology section of the Russian Bioethics Committee.

## Author Contributions

BS and KS conceptualized the study. BS, KS, NV, and GK contributed to the study methodology. AB, TS, and GK validated the study. KS and IL undertook the formal analysis. KS, IP, and NV investigated the study. BS and GK contributed to the resources. KS, IP, and NV undertook the data curation. KS was responsible for the original draft preparation. IP, NV, AB, TS, GK, and BS were responsible for the review and editing of the original draft. IP visualized the study. BS supervised the study and responsible for the funding acquisition. All authors have approved the final version of the manuscript and agreed to be accountable for all aspects of the work. All persons designated as authors qualify for authorship, and all those who qualify for authorship are listed.

## Conflict of Interest

The authors declare that the research was conducted in the absence of any commercial or financial relationships that could be construed as a potential conflict of interest.
